# Unexpected Discoveries Should Be Reconsidered in Science—A Look to the Past?

**DOI:** 10.3390/ijms20163973

**Published:** 2019-08-15

**Authors:** Alberto Foletti, Stefano Fais

**Affiliations:** 1 Clinical Biophysics International Research Group, 6900 Lugano, Switzerland; 2 Institute of Translational Pharmacology, National Research Council-CNR, 00133 Rome, Italy; 3 Department of Oncology and Molecular Medicine, National Institute of Health, 00133 Rome, Italy

## Abstract

From the past, we know how much “serendipity” has played a pivotal role in scientific discoveries. The definition of serendipity implies the finding of one thing while looking for something else. The most known example of this is the discovery of penicillin. Fleming was studying “Staphylococcus influenzae” when one of his culture plates became contaminated and developed a mold that created a bacteria-free circle. Then he found within the mold, a substance that proved to be very active against the vast majority of bacteria infecting human beings. Serendipity had a key role in the discovery of a wide panel of psychotropic drugs as well, including aniline purple, lysergic acid diethylamide, meprobamate, chlorpromazine, and imipramine. Actually, many recent studies support a step back in current strategies that could lead to new discoveries in science. This change should seriously consider the idea that to further focus research project milestones that are already too focused could be a mistake. How can you observe something that others did not realize before you? Probably, one pivotal requirement is that you pay a high level of attention on what is occurring all around you. But this is not entirely enough, since, specifically talking about scientific discoveries, you should have your mind sufficiently unbiased from mainstream infrastructures, which normally make you extremely focused on a particular endpoint without paying attention to potential “unexpected discoveries”. Research in medicine should probably come back to the age of innocence and avoid the age of mainstream reports that do not contribute to real advances in the curing of human diseases. Max Planck said “Science progresses not because scientists change their minds, but rather because scientists attached to erroneous views die, and are replaced”, and Otto Warburg used the same words when he realized the lack of acceptance of his ideas. This editorial proposes a series of examples showing, in a practical way, how unfocused research may contribute to very important discoveries in science.

For a couple of decades, Stefano Fais has been involvement in cancer research. However, before arriving at cancer research he was involved in research on mucosal immunology of the gut [1,2,3,4,5,6,7,8,9], neuroimmunology [10,11,12], AIDS research [13,14,15], animal models [16,17,18], and the involvement of the cytoskeleton in subcellular pathophysiology [19,20,21,22,23,24,25]. He also had long-standing clinical experience, which led to participation in clinical trials in both diagnostic and therapeutic areas [26,27,28]. However, the aim here is not to present a self-celebration list, but rather to summarize the journey of how he became involved in oncology. He arrived at that moment with extensive experience in research in medicine and, therefore, without the superstructure of the experimental oncology environment. In essence, he wanted to understand where research in oncology was at through the eyes of a child. Actually, he was somewhat deluded by the lack of change in therapy and scientific data, particularly in the area of experimental oncology. In this regard, while studies were frequently published in top-tier journals, evidence was often considered weak and rarely translated into clinical use. Unfortunately, however, this was not much different for all diseases that were hardly curable three or four decades ago and are still not curable today (e.g., chronic diseases), regardless of the high level of investment from both public and private sectors. On this basis, we may wonder if something could actually be wrong with the way research is currently undertaken worldwide. This thought has been supported by clear evidence that the epochal discoveries in science were derived from a completely different approach that is without programs on practical targets. The current mainstream research approach examines extremely small focused targets. However, to be honest, results derived from this approach are often disappointing and it is difficult to comment on them.

First of all, we would like to recount some stories of Nobel Prize winners that arrived at their discoveries without a precise idea of what they were looking for.

These famous names in Science include Ilya Ilyich Mechnikov, Otto Heinrich Warburg, Alexander Fleming, and more recently, Barry Marshall and Robin Warren. 

In 1882 Mechnikov first demonstrated a novel process when he discovered in the larvae of starfish, mobile cells that, he hypothesized, could serve as part of the defenses of these organisms. To test this hypothesis, he introduced small thorns from a tangerine tree, which had been prepared as a Christmas tree for his children, into the larvae. Next morning, he found the thorns surrounded by the mobile cells. From this unexpected finding, he thought that when inflammation occurs in animals that have a blood vascular system, the white cells may be directed from the blood vessels to the site of inflammation to protect the body from the attack of foreign agents such as bacteria, using ingestion and digestion of the foreign bodies as a key mechanism for their protective action. He called the cell that surrounds and kills pathogens “phagocyte” [29]. His theory, that certain white blood cells could engulf and destroy harmful bodies such as bacteria, was met with skepticism in the academic world, which included eminent scientists such as Louis Pasteur. At the time, most bacteriologists believed that white blood cells ingested pathogens and then spread them further throughout the body. His discovery of these phagocytes ultimately won him the Nobel Prize in 1908. In 1887, he observed that leukocytes isolated from the blood of different animals were attracted towards certain bacteria. However, Mechnikov’s early observation represented the background for studies that defined a critical mechanism by which bacteria attract leukocytes to initiate and direct the innate immune response against infectious agents through opsonins [30]. It is probable that Mechnikov also contributed to the hypothesis of the role of lactic acid bacteria in curing infectious diseases of the gut, and was thus also a pioneer of the use of pro-biotics [31].

In 1928, Alexander Fleming was studying “Staphylococcus influenzae” when one of his culture plates became contaminated and developed a mold that created a bacteria-free circle. Then he discovered, by chance, within the mold, a substance that proved to be very active against the vast majority of the bacteria infecting humans [32].

In 1924, the biochemist and Nobel Laureate Otto Heinrich Warburg postulated that cancer cells differ from normal healthy cells in their metabolism [33]. The majority of normal cells use the Krebs cycle, which requires oxygen to convert glucose to energy. Warburg presented evidence that cancer cells fermentate sugar, either in the presence or in the absence of oxygen. At the end of this process, cancer cells produce lactic acid that is the major determinant of the acidic extracellular cellular microenvironment, representing a common phenotype of virtually all cancers [34]. In fact, Warburg reported that cancer cells maintain a lower pH, as low as 6.0, due to lactic acid production and elevated CO_2_. Despite the unbelievable value of Otto Warbur’s discovery, most of his peers did not take his hypothesis seriously (except for the Nobelist and co-discoverer of vitamin C, Albert Szent-Gyorgyi). Actually, so far, the Warburg Hypothesis has been almost completely ignored. A take home message we want to preserve from Warburg’s hypothesis is that, while cancer, above all other diseases, has countless secondary causes (almost anything can cause cancer), there is probably only one common prime cause, “the replacement of the oxygen respiration (oxidation of sugar) in normal body cells with sugar fermentation”, with production and accumulation of lactic acid leading to a progressive acidification of the tumor microenvironment.

Continuing Warburg’s discovery, Stefano Fais’s group has published a series of articles showing that a class of drugs called proton pump inhibitors (PPIs) were able to revert tumor drug resistance due to the acidic tumor microenvironment’s ability to neutralize the vast majority of drugs, being weak bases [35,36], to kill cancer cells and inhibit cancer growth by inhibiting a mechanism that cancer cells use to avoid intracellular acidification [37,38,39,40,41,42,43,44,45], and also to increase the efficacy of immunotherapies due to the weakness of immune cells at the low tumor pH [46]. These data were supported by results obtained with sodium bicarbonate [47] and more recently supported by clinical data [48]. These preclinical data also led to clinical studies providing very encouraging results showing that PPIs improve the effectiveness of standard chemotherapy [49,50,51,52,53,54]. These clinical data have been in part confirmed by two retrospective studies in different cancer patients [55,56]. Other pre-clinical studies obtained in in vivo models have shown that either the addition of buffers to daily water supply may control cancer growth [57], or water alkalinization may prevent prostate cancer [58], both theories supporting a previous study showing that simply adding sodium bicarbonate to water provided a 100% prevention of prostate cancer development in TRAMP mice [59]. All these data contributed to an international consensus [60,61,62,63], and some papers are supporting a repositioning of proton pump inhibitors in cancer treatment [64,65,66].

Nearly 35 years ago, two Australian physicians made a discovery that was initially ignored by the medical community. Barry Marshall and Robin Warren claimed that stomach ulcers were caused by a bacterium called *Helicobacter pylori* and not by excessive acidity in the gastric environment [67].

To test his theory, Marshall ingested a *Helicobacter pylori* preparation, in turn provoking a documented formation of ulcers in his stomach. He then treated these provoked ulcers with a combination of antibiotics and anti-acidic drugs that led to the healing of the gastric lesions. After this experiment, an NIH Consensus Development Conference Statement concluded that there was indeed a strong association between ulcers and *Helicobacter pylori* and recommended using antibiotics as the preferred treatment of Peptic Ulcer Disease [68]. At that time, only a small fraction of patients with ulcers underwent antibiotics treatment. By 1996, the Food and Drug Administration approved the antibiotic treatment for Peptic Ulcer Disease in association, of course, with anti-acidic drugs; the so called “eradicating treatment of Peptic Ulcer Disease”. Today, the “eradicating treatment” is a standard therapy for both gastric and duodenal ulcers. It should be emphasized that this finding was obtained without specific project-derived funds, but with a careful look at a standard bioptic sample preparation. After the pivotal experiment by ingestion of *H. pylori* was published in the *Medical Journal of Australia* [69], Barry Marshall and Robin Warren received the Nobel Prize in 2005.

We often quote and discuss an article that did not appear in a scientific journal, but in the Financial Times, 2008 [70]. Among the many interesting thoughts of the authors were: “What went wrong? The answer, we suggest, is the mis-measure of uncertainty, as academic researchers underestimated the fragility of their scientific knowledge, while pharmaceuticals executives overestimated their ability to domesticate scientific research.”; and “For all the breathless headlines proclaiming breakthrough discoveries, the truth is that we still do not understand what causes most diseases. Even when we can identify a responsible gene or implicate an important mutation, we have made only limited progress in turning these results into treatments.”; and finally “Medical research is particularly hampered by the scarcity of good animal models for most human disease, as well as by the tendency of academic science to focus on the “bits and pieces” of life—DNA, proteins, cultured cells—rather than on the integrative analysis of entire organisms, which can be more difficult to study.” The authors have been visionary, because I think there is something to be explained in what is occurring in the research and development of new and effective drugs against major diseases. The results are delusive actually, in as much as the so called “new drugs” inspired by the idea of Paul Erlich’s “magic bullet” are very expensive and not too convincing in terms of effectiveness. Evidence is provided by articles showing the clinical ineffectiveness of new anti-cancer drugs, but also by the decision of Big Pharma to stop, for instance, research on anti-Alzheimer or anti-Parkinson molecules because nothing substantial has been discovered so far.

Psycho-Neuro-Endocrine-Immunology (PNEI) is a scientific field of study that investigates the real time permanent cross-links among three well-defined compartments of our body, such as the nervous, the endocrine and the immune systems, that together may influence our psychology. A paradigm of this integrated and continuous cross-talk within our body is that it highly influences our health. The PNEI innovative medical approach implies that our body works as an all in one system. Evidence that, from the beginning, supported the existence of PNEI showed that immune cells release hormones and also that endocrine cells release neurotransmitters that may influence the immune response; the so called “bidirectional cross-talk” between the psycho neuroendocrine and immune systems [71]. One outcome of PNEI is the low dose medicine [72], which was able to allow the researchers to design innovative therapeutic strategies for the treatment of skin diseases based on rebalancing the immune responses [73].

There is evidence that an aberrant tumor microenvironment facilitates cancer development, progression, and responses to treatment [74,75]. Laboratory models have focused the malignant epithelium, while an increasing number of studies over the past 20 years have begun to examine the role of interactions between tumor epithelium and stroma in the development of breast and other cancers [76]. It is unclear whether existing models can provide predictive information on the efficacy of small molecules to test in human clinical trials. Therefore, considerable investment will be needed to overcome difficulties addressing host-tumor interactions for the benefit of the cancer patient.

Another non-mainstream approach includes the somatic mutation theory, an approach that is often neglected. The so-called somatic mutation theory can be explained by the fact that “A somatic cell serially accumulates genetic damage, eventually reaching a point at which it decouples from the organism’s regulatory systems and embarks on its own agenda” [77].

Furthermore, it underlines the fact that the majority of tumors are not of genetic origin (somatic mutation theory) [77] but can be explained with the Tissue Organization Field Theory proposed by Sonnenschein and Soto [78]. This is a good example of how the dominant paradigm influences the world of research [79], defining hallmarks within which to conceive, finance, and conduct research [80,81]. 

An additional example of non-mainstream treatment related to rheumatoid arthritis therapy is found in the use of bioelectric modulation of the body’s inflammatory responses through the neurovegetative system and the consequent neuro-immuno-modulation response [82].

A similar approach that lends itself well to the description of “Non-mainstream approach in science discoveries” is the area of biophysical medicine, a less well known field that is gaining significant interest in different clinical settings. Biophysical treatment is a novel and integrative emerging tool in clinical practice, and a number of clinical studies have already documented potential beneficial effects from this approach in the management of pain [83,84,85], as well as in the management of psoriasis [86], minor anxiety and depressive disorders [87], and chronic kidney disease [88]. It is thought that biophysical treatment can exert clinical effects through a resonance effect [89]. Resonance occurs between therapeutically delivered electromagnetic signals, endogenous or exogenous, and target tissues, organs, and/or the entire organism [90,91,92], allowing the achievement of both local and systemic effects at the same time [93].

In summary, it would appear that cancer may be regarded as a default state of cells that are stressed or affected in a particular way, such as by the aging process or exposure to carcinogens. Tumor development may represent the reversion to an ancestral phenotype [94]. Unfortunately, current research still tends to be too specific, with study outcomes on specific targets, thus hampering the possibility of the significant discoveries in medicine that our grand predecessors made.

Lastly, a couple of other example of non-mainstream discoveries are: (i) The new idea of tumor cannibalism, which was described in tumor samples at the end of 1800 [95,96] and has been reappraised by two different groups with two different approaches and re-named “cell-in-cell phenomena” [97], and can definitively be considered as a common phenotype of all malignant tumors; and (ii) The use of extracellular vesicles of a nanosize (exosomes) as a circulating tumor marker, with increased exosome release and plasmatic levels being a distinguishing features of malignancy [98,99,100,101]. This led to provocative reviews raising a new role of exosomes in metastatic dissemination, and as clear evidence of the high level of toxicity of the tumor microenvironment [102,103]. Some new anti-tumor strategies are taking place based on anti-oxidant treatments, such as the use of retinoic acid [104] or ascorbic acid [105] in the treatment of patients with leukemia and lymphomas; more recently, preclinical in vivo evidence has shown that daily treatment with fermented papaya may control the growth of a very aggressive melanoma [106]. All this evidence, together with the anti-acidic treatments [64], suggests that non-aggressive therapies may well work as new types of cancer treatment and could probably support important changes in anti-cancer strategies. Most of all, they suggest that a non-mainstream approach remains an important and fruitful old/new strategy in science discovery [107].
